# Bacteriophage Encapsulation in pH-Responsive Core-Shell Capsules as an Animal Feed Additive

**DOI:** 10.3390/v13061131

**Published:** 2021-06-11

**Authors:** Kerry Richards, Danish J. Malik

**Affiliations:** Department of Chemical Engineering, Loughborough University, Loughborough LE11 3TU, UK; k.richards@lboro.ac.uk

**Keywords:** antibiotic resistance, animal feed, bacteriophages, biocontrol, controlled release, core-shell capsules, microbiome engineering

## Abstract

Increasing antibiotic resistance in bacteria that cause zoonotic infections is a major problem for farmers rearing animals for food as well as for consumers who eat the contaminated meat resulting in food-borne infections. Bacteriophages incorporated in animal feed may help reduce carriage and infections in animals including chickens and pigs. There are, however, unmet challenges in protecting phages from processing stresses e.g., during animal feed pelleting operations and during transit of phages through the acidic gastric environment. Core-shell capsules were produced using a concentric nozzle and commercially available encapsulation equipment to fabricate capsules with phages formulated in an oil-in-water microemulsion in the core. pH-responsive capsules released the encapsulated phage cargo within 10–30 min triggered by changes in local environmental pH typically found in the lower gastrointestinal (GI) tract of animals. Acid stability of phages exposed to pH values as low as pH 1 was demonstrated. Encapsulated phages were able to withstand exposure to 95 °C wet heat thermal stress for up to 120 s, conditions typically encountered during feed pellet extrusion processing. Free phages were inactivated within 15 s under these conditions. The present study demonstrates that encapsulation of bacteriophages in core-shell pH-responsive capsules with water-in-oil emulsified phages in the core significantly improves phage viability upon exposure to processing and environmental stresses that require consideration during production of animal feed and application in animals for biocontrol. The results from this study should help guide future development of phage formulations suitable for use in animal feed for animal biocontrol applications.

## 1. Introduction

Incidences of severe gastrointestinal (GI) bacterial infections in humans are rising due to the emergence of antibiotic resistance in bacteria. The animal feed industry relies on antibiotics for zoonotic disease control and animal welfare; thus, antibiotics are used in significant quantities. Animals are often housed in cramped stressed conditions at high densities (e.g., in chicken meat production) with disease rapidly spreading between flocks [1]. Common farming practices include antibiotic treatment of all animals within the group after the clinical onset of symptoms across a minority of the population. Antibiotic administration is routinely provided through animal feed/water, enabling entire groups of animals to be treated with maximum time efficiency. However, with sub-therapeutic doses facilitating the rise of antibiotic resistance, animals could be acting as a reservoir for untreatable bacterial infections, transmitting through the food chain and causing outbreaks of infections [2]. Sub-therapeutic concentrations of 16 antimicrobials were tested in swine against four enteric pathogens (*Salmonella enterica* serotype *Typhimurium*, *Yersinia enterocolitica*, *Shigella flexneri* and *Proteus mirabilis*). Ten of the 16 antimicrobials amplified resistance or virulence gene transfer, demonstrating the urgent present need for an antibiotic alternative to prevent long-term health implications for society.

The most common zoonotic pathogens are *E. coli* (STEC), *Salmonella* spp., *Campylobacter* and *Listeria monocytogenes*, all of which are becoming multi-drug resistant [3]. Hygiene methods attempt to control zoonotic pathogens through the food chain, yet economic interests prevail with antibiotics added to animal feed for improved yields and productivity in the meat sector [4]. The pharmacokinetic parameters (metabolism, adsorption and excretion) determine the possibility of antibiotic residues to remain in animal products, possibly posing a risk to human health through intestinal microbial imbalance [5]. An estimated 63,000 to 240,000 metric tonnes of antibiotics are used in agriculture each year, increasing with the rise of meat consumption in emerging economies [6]. Livestock production is growing exponentially to meet the current population demand; consequently, the issue of resistance will only worsen if alternatives to broad spectrum antibiotics are not urgently explored. A method for bacterial disease control, which is effective, low cost and will target only the pathogenic bacteria, is necessary for the sustainable future of the animal food production industry.

The World Health Organisation has called for antibiotic alternatives to be implemented into animal feed to control the exponential emergence of antibiotic resistant mutants [7]. Bacteriophages (“phages”) are viruses which can infect and selectively lyse specific bacterial strains within their host range without disruption of the wider microbiota. Phage amplification relies on their adsorption to phage binding receptors in the bacterial cell wall, insertion of phage DNA and hijacking of bacterial replication systems resulting in bacterial cell lysis and release of phage progeny [8]. Phages are promising drug candidates for bacterial control due to their bacterial specificity and their safety profile. The ubiquitous nature of phages allows environmental isolation of virulent phages against pathogenic antibiotic resistant bacterial strains, notably from sewage and soil [9,10]. A significant advantage of using phages as biocontrol agents for foodborne pathogens is their constant evolution, an aspect not achievable with antibiotics which may help in overcoming the issue of phage resistance. Many studies have reported successful results showing reduction in bacterial loads using phage-based approaches [11,12,13]. Continuous phage production has the potential to produce industrial quantities of phages in an economic manner. Implementation of continuous phage production was recently demonstrated using a series of bioreactors decoupling host propagation from phage predation allowing scalable economic production of low-cost phages using industrial fermentation process technologies [14]. Therefore, the availability of phages as supplements in animal feed instead of using antibiotics is a real possibility. Bacterial control in eggs through the utilisation of phages has also been evaluated recently [15]. A *Salmonella-*specific Podoviridae (Pu20) showed lytic activity against two bacterial strains when tested in egg white and yolk. Pu20 had heat and pH tolerance with short incubation periods [15]. The application of bacteriophages in animal feed has not yet been investigated in any great detail and merits greater scrutiny.

Animal feed pellet production includes hot extrusion (using steam) of the feed meal to form the feed pellets. Feed extrusion includes feed constituent mixing, addition of wet heat to add moisture and allow extrusion through pellet forming dies typically a few mm in diameter to form the feed pellets [16]. Continuous production lines often operate with the input of ingredients and output of materials balanced to maintain high efficiency. Phage incorporation in the animal feed pelleting process requires consideration of the processing stresses that the phages will be subjected to, specifically wet heat and extrusion stresses.

Barriers to phage application within animal feed were outlined in a recent review with phage delivery (concentration and dosage) and method of application highlighted as areas requiring attention [17]. Not only must the phage survive the production process, but during treatment exposure to degradative enzymes and phage stability upon exposure to gastric acidity remain a challenge [18]. Encapsulation of phages could afford them protection during the feed manufacture process, creating a microenvironment for the phages during transit to the desired target site. A bioencapsulation method by which artemia (brine shrimp) were enriched with *Edwardsiella tarda* phage (ETP-1) reported successful in vivo results [19]. The artemia were subsequently fed to adult zebra fish and phage dissemination over time was measured. Phages were found to be present in the spleen, gut, kidney, and liver of the zebra fish whilst continually being fed ETP-1 enriched artemia, reductions were observed after feeding was suspended. Furthermore, microencapsulation of a 16-phage cocktail in alginate beads against *S. Typhimurium* was shown to be effective when administered via oral gavage to market-weight pigs. Significant reductions in bacterial load were observed in the caecum compared with the control samples [20]. Encapsulation of phages for oral delivery to poultry and livestock in animal feed could provide phages protection from stresses encountered in the gastrointestinal tract of the animals allowing targeted release of high phage doses at the site of infection or for prophylactic use to reduce carriage and transmission. 

The aim of the present study was to develop a novel core-shell encapsulation process whereby phages were incorporated in a water-in-oil emulsion which subsequently formed the core of the core-shell capsules with the shell made-up of a pH-responsive polymer. This is the first time that such as approach has been evaluated to protect phages from animal feed pellet forming process stresses including exposure to wet heat whilst also affording the encapsulated phages protection from gastric acidity encountered during gastrointestinal transit to the caeca where the phage cargo is to be released targeting pathogenic bacteria residing therein.

## 2. Materials and Methods

### 2.1. Escherichia coli Growth Conditions and Bacteriophage T3 Propagation

*Escherichia coli* strain ATCC11303 and its lytic phage T3 (ATCC11303-B3, family Podoviridae) were sourced from LGC Standards (Teddington, Middlesex, UK). Single colonies of *E. coli* were selected by streaking onto LB agar (25 g/L LB broth Miller, Fisher Scientific U.K., Loughborough, UK) with 1.5 *w*/*v*% Bacteriological Agar No. 1 (Oxoid, Basingstoke, UK) and incubated overnight at 37 °C. A single *E. coli* colony was added to a sterile 200 mL flask containing 25 mL of LB broth and the flask was incubated in the shaking incubator (Certomat^®^ BS-1) overnight at 37 °C 150 RPM. After incubation, the overnight culture was centrifuged at 4500× *g* for 10 min, the supernatant was aspirated and the pellet was resuspended in 5 mL SM buffer (100 mM NaCl, 8 mM MgSO_4_, 50 mM Tris-HCl, pH adjusted to 7.5 using 4 M HCl) for refrigerated storage up to two weeks.

To amplify bacteriophage T3, a fresh *E. coli* culture with a starting OD_600nm_ value of 0.05 was prepared. The absorbance (OD_600nm_) was measured until the optical density value reached 0.2 (corresponding to 10^7^ CFU/mL). Subsequently, the culture was inoculated with phage T3 at a multiplicity of infection (MOI) value of 0.001. Immediately after T3 infection, ammonium sulphate (Fisher Scientific U.K.) at a working concentration of 25 mM was added to the culture. The flask was incubated, and the absorbance was monitored until the optical density decreased to ~0.05. The T3 culture was then pipetted into a sterile centrifuge tube (50 mL Falcon, Fisher Scientific U.K.) and centrifuged 4500× *g* for 10 min, the supernatant was then filtered using a 0.45 µm membrane (Stericup Vacuum Filtration System, Fisher Scientific U.K.). Following this, the phage was concentrated using a 100 kDa ultrafiltration Amicon tube (Fisher Scientific U.K.) and diafiltration performed with five repeated washes with SM buffer (100 mM NaCl, 8 mM MgSO_4_, 50 mM Tris-HCl, pH 7.5) and repeated centrifuge cycles of 1000× *g* for 10 min.

Final phage titres were enumerated using the standard plaque assay [21]. For each plate, 5 mL of LB top agar (LB broth, Fisher Scientific U.K. with 0.5% Bacteriological Agar No. 1, Oxoid, Basingstoke, UK) was combined with 5 mL salt solution (400 mM MgCl_2_ and 100 mM CaCl_2_) in a sterile centrifuge tube. 10 µL of *E. coli* culture was added to the agar solution after cooling and poured over a LB agar plate. The phage sample was serially diluted in LB broth using a sterile 96 well plate from dilution factor 10^−1^ to 10^−8^. 10 µL of each dilution sample was spotted in quadruplicate and incubated overnight at 30 °C or for 4 h at 37 °C.

### 2.2. Chemical Reagents

Eudragit polymer S100 is a methyl methacrylate co-methacrylic acid copolymer purchased from Evonik (Essen, Germany). Medium viscosity alginate was purchased from Sigma Aldrich (Gillingham, Dorset, UK). Calcium chloride and tween 20 were purchased from Fisher Scientific U.K. Miglyol 840 is a propylene glycol diester of saturated plant fatty acids purchased from Safic Alcan UK (Warrington, Cheshire, UK). Polyglycerol polyricinoleate (PGPR) was purchased from Aston Chemicals (Aylesbury, Buckinghamshire, UK) and is an emulsifier made from glycerol and fatty acids.

### 2.3. Preparation of Solutions

The oil phase was produced by preparing a solution of Miglyol with the addition of 5% PGPR (*w*/*w*) to lower the interfacial tension between water and oil. The aqueous phase was composed of 10% Eudragit polymer (*w*/*v*) dissolved in an alkaline solution, typically produced in 40 mL batches with 3 ml of 4 M NaOH, 37 mL dH_2_O and 4 g Eudragit S100. This solution was mixed with a magnetic stirring bar until the solution appeared clear. Subsequently, medium viscosity alginate was added at a final working concentration of 1% (*w*/*v*) and mixed with a magnetic stirring bar at 60 °C until being completely dissolved. A solution of 40 mL dH_2_O was produced with the addition of 0.4 g (1% *w*/*v*) alginate for use in experiments with an outer shell of alginate only. Immediately before the production process commenced, T3 at a concentration of approximately ~10^9^ PFU/mL was added to the oil phase at a ratio of 1:10, respectively, and emulsified using a homogeniser (IKA T25) for 15 s on the slowest speed to form a water-in-oil emulsion.

### 2.4. Droplet Generation Using a Concentric Nozzle Buchi B-390 Encapsulator

**Figure 1:**
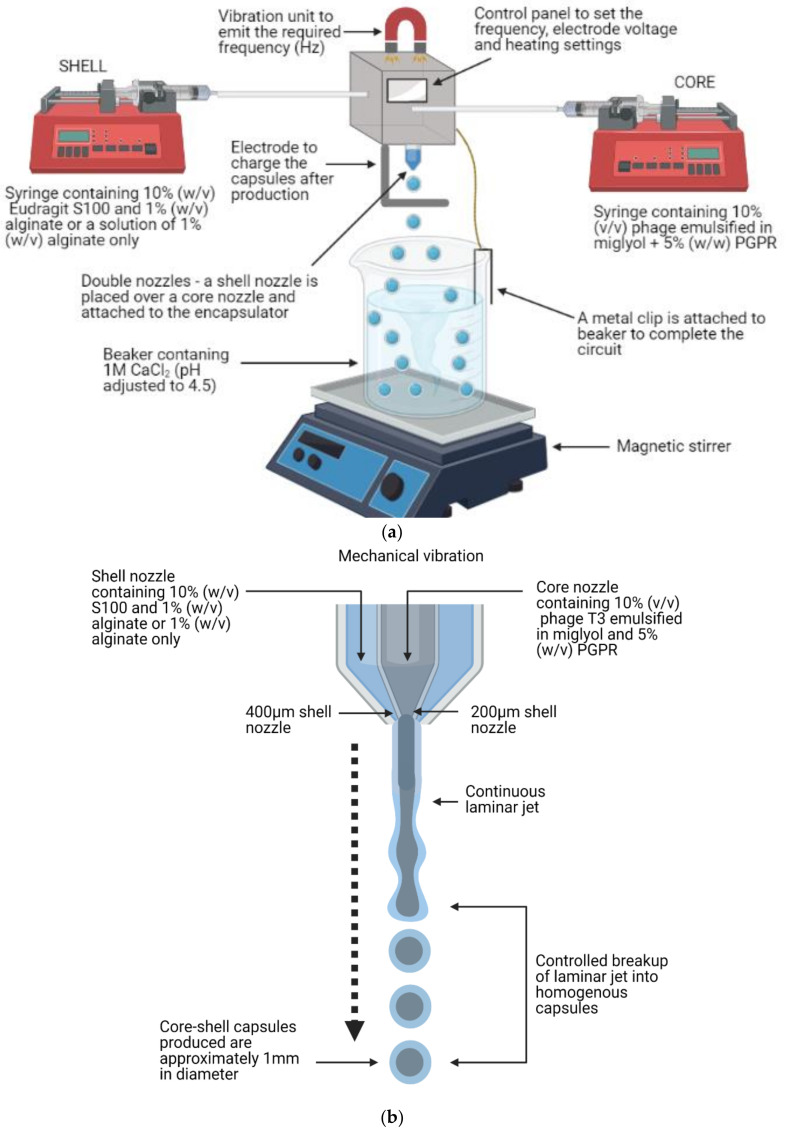

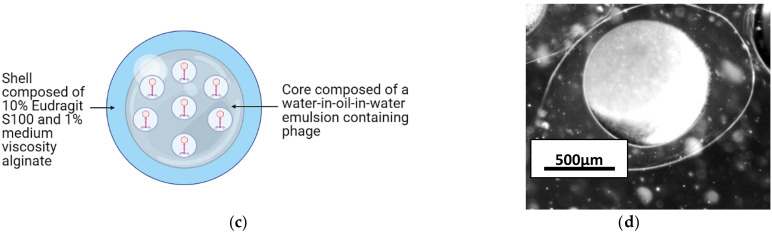
Schematic representation of the core-shell capsule production process using the Buchi encapsulator. (**a**) The ‘core’ syringe contained an emulsion composed of T3 phages, Miglyol +5% (*v*/*v*) polyglycerol polyricinoleate (PGPR) and was set to a flow rate 3× lower than the shell syringe. The ‘shell’ syringe contained a solution of 10% (*w*/*v*) S100 and 1% (*w*/*v*) medium viscosity alginate or a solution of 1% (*w*/*v*) alginate only. An outer (shell) and an inner (core) nozzle were used, sealed with a rubber gasket and the vibration unit (magnet) was placed above the nozzle head. The frequency and electrode parameters were set and maintained at a constant setting before the syringe pumps were started. The capsules were collected in a beaker located beneath the nozzle containing 1 M CaCl_2_ with gentle agitation using a magnetic stirrer bar at 100 RPM. (**b**) Representation of the laminar jet production process by which the nozzle forms a droplet within the shell nozzle jet. (**c**) Schematic diagram of the core-shell capsules including the phage contained in a water-in-oil emulsion and S100/alginate outer shell. (**d**) Optical image of the capsules before alginate crosslinking and polymer protonation. The water-in-oil core was surrounded by an S100/ alginate shell, captured using a ×10 magnification lens. Created with BioRender.com.

The Buchi B-390 Encapsulator (Buchi, New Market, UK) was assembled using the 200 µm inner nozzle (core) and the 400 µm outer nozzle (shell) (Figure 1a). Two Harvard syringe pumps equipped with 20 mL syringes were used. Each syringe independently controlled the flow to the core and shell nozzles. The syringe connected to the shell nozzle contained 20 mL of 10% S100 (*w*/*v*) and 1% medium viscosity alginate (*w*/*v*) with flow rate set at 12 mL/min. For the alginate-only capsules the syringe connected to the shell nozzle contained 20 mL 1% alginate (*w*/*v*) and the flow rate was set at 12 mL/min. The syringe connected to the core nozzle contained 10 mL of the T3 water-in-oil emulsion previously prepared and flow rate set to 4 ml/min. The optimal operation parameters for particle production were initially established (Frequency: 1000 Hz, electrode 1000 V) after a series of experiments. Once a resonator induced jet break-up regime had formed, the capsules were allowed to drop directly into 100 mL of 1 M CaCl_2_ (pH adjusted to 4.5) which was kept gently stirred at 100 RPM (Figure 1b). Typically, 5 ml of the ‘core’ was passed through the syringe, and 15 mL of the ‘shell’ per batch.

### 2.5. Capsule Size Characterisation

Laser diffraction was selected for measuring the size of the alginate only capsules which were less than 900µm in size (LS coulter 130). Initially, a solution of 2% tween 20 (pH 4.5) was used to fill the measurement. The background was then set and the obscuration was monitored to ensure this remained at 0%. Alginate capsules were subsequently added until the obscuration reached between 8–12%, following which the measurement program was started. Three runs of 60 s were completed in which results were generated and averaged using Microsoft Excel. Glass beads were used as a control before the capsules were measured. For size analysis of S100 + alginate capsules, ImageJ was utilised in which a sample size of 20 capsules were measured and subsequently analysed using Microsoft Excel.

### 2.6. Core-Shell Capsule Production

After the capsules were formed, they were solidified using alginate crosslinking with CaCl_2_. We added 400 mL of 1 M CaCl_2_ (pH adjusted to 4.5) to the original 100 mL after capsule production. This ensured the capsules did not coalesce before they had solidified. The capsules were stirred at 100 RPM using axial mixing from a three bladed impellor for 2 h at a controlled temperature of 37 °C. After this, the beaker was removed from the stirrer and the capsules settled to the base, thus the CaCl_2_ solution could be aspirated. The capsules were then washed with 200 mL 2% tween 20 (pH 4.5) to remove any residual CaCl_2_ ions. After this, the S100 + Alginate capsules were resuspended in 500 mL 2% tween 20 (pH 1.5) and stirred, using the same impellor, at 100 RPM for 2 h at a controlled temperature of 37 °C. The alginate-only capsules were purified immediately after the CaCl_2_ was removed, without the 2% tween step. To purify the final capsules, first the 2% tween was carefully aspirated using a 10 mL pipette to ensure no capsules were discarded during the buffer removal step. Subsequently, the capsules were then transferred to a vacuum filter (0.45 µm, Fisher Scientific U.K.) and any remaining buffer was removed, leaving dry capsules. The final capsules were stored in sealed falcon tubes in the refrigerator at 4 °C.

### 2.7. Testing the Efficacy of Encapsulated T3 in Simulated Gastrointestinal Conditions

The phages were released from the core-shell capsules by exposure to Sorensen’s buffer (0.2 M sodium phosphate and 0.2 M sodium phosphate dibasic) at pH 7.5. The capsules were suspended for 2 h to dissolve at 37 °C. Time points were taken every 10 min to understand the kinetics of release. Final phage counts were enumerated through the double layer method [21]. To complete this, 20 µL of supernatant was added to 180 µL of SM buffer and diluted 10-fold to 10^−8^*. E. coli* cultures were used to produce a bacterial lawn in which the samples were spotted in quadruplicate. Three batches of capsules were produced, and the final phage titres were averaged to determine the final recovery yield.

### 2.8. Core-Shell Capsule Stability after Exposure to Simulated Gastric Fluid

The acid stability of T3 phages before encapsulation was initially investigated to demonstrate the need for encapsulation. We added 100 µL of T3 phage in SM buffer at a titre of approximately ~10^10^ PFU/mL to 900 µL of acidic buffer (0.2 M NaCl adjusted using 4 M HCl). pH values of 1, 1.5, 2, 2.5, 3, 4, 5 and 6 were tested for up to 48 h. The phages in buffer were incubated at 37 °C and time points were taken throughout the testing period for phage enumeration using the standard plaque assay.

To determine the acid stability of encapsulated T3, 0.1 g of T3 containing core-shell capsules were added to a sterile bijoux (7 mL, Fisher Scientific U.K.) along with 1 ml of SGF (0.2M NaCl) at pH 1, 1.5, 2 and 2.5. The buffers used for acid exposure were adjusted using 1M HCl and the final pH was measured with a pH meter (Jenway 3510 pH Meter). The bijoux containing core-shell capsules and SGF was incubated at 37 °C in a shaking incubator (Certomat, BS-1, Sartorius, UK) for 2 h. Subsequently, the capsules settled to the base of the bijoux and the supernatant was carefully aspirated. The capsules were then resuspended in 1ml of Sorensen’s buffer (pH 7.5) and incubated further for 2 h at 37 °C. Phage titres were confirmed using the standard plaque double overlay assay (described previously).

To confirm the Eudragit S100 had not protonated further during the acid exposure, the release kinetics of the capsules after acid exposure were determined as outlined above. The capsules were released in Sorensen’s buffer (pH 7.5) after the acidic buffer was aspirated and time points were taken for a 2 h testing period. The phage titres were then enumerated using the standard plaque assay.

### 2.9. Thermal Treatment of Free and Encapsulated T3

The thermal tolerance of T3 phage, before encapsulation, was investigated using a heating block (Stuart Scientific Block Heater) and metal crucibles. A thermocouple (RS Pro, RS-41) was placed in the heating block to confirm the internal temperature. 1ml of T3 phage at a titre of approximately ~10^9^ PFU/mL was added to each metal crucible and placed into the heating block. Every 5s a sample was taken from the heating block for serial dilution. Samples were taken for 20s of thermal treatment at 75 °C, 85 °C or 95 °C and the phage titres were enumerated using the plaque assay.

Subsequently, the core-shell capsules were tested for the heat stability they offer in comparison. Typically, 0.1 g of capsules were weighed into metal crucibles and placed in the heating block set to 75 °C, 85 °C or 95 °C. At each time point, the crucibles were removed from the heating block and submerged into an ice bath. Time points were taken at 30 s, 60 s, 90 s and 120 s. After the capsules were exposed to heat and then cooled, they were collected in sterile bijoux containers. 1 ml of Sorensen’s buffer (pH 7.5) was added to enable capsule dissolution. 0.1 g of capsules from the same batch, which were not exposed to any thermal treatment, were used as a control. 3 batches were produced and 3 samples from each batch were used for each time point.

After heat exposure, the core-shell capsules were tested for release kinetics to determine if the thermal treatment had affected the polymer’s pH-responsive characteristics. 0.1 g of capsules were exposed to 95 °C for 120 s before dissolution in 1mL Sorensen’s buffer (pH 7.5). Time points were taken every 10 min for 1 h and every 30 min for the remaining hour. The samples were serially diluted and the release profile was determined through plaque enumeration. Heat exposed capsules were also exposed to pH 1 for 2 h to ensure the capsule integrity had not been compromised during the heating process. The capsules were then exposed to Sorensen’s buffer to release the T3 phages and phage enumeration carried out as outlined previously.

### 2.10. Simulation of the Pellting Extrusion Process to Investigate the Effect of Shear on Capsule Integrity

A simulated extrusion model was designed using a syringe pump and dough mixture to investigate the integrity of the core-shell capsules after extruding. A dough mixture composed of 40% (*w*/*w*) flour, 25% (*w*/*w*) oil and 15% (*w*/*w*) water was used. Core-shell capsules were added to the dough mixture at a final mass fraction of 5% (*w*/*w*). The capsule-dough mixture was loaded into a syringe with an outlet diameter of 3 mm and placed in a Harvard Elite syringe pump. The syringe pump was set to a flow rate of 15 mL/min and the dough mixture was extruded through the syringe to produce pellets. After pellet production, the extruded capsules were harvested, and their integrity was visually assessed. To examine any damage to the capsules, one portion of the batch was exposed to Sorensen’s buffer (pH 7.5) and the remainder exposed to pH 1 for 2 h before releasing in Sorensen’s buffer (pH 7.5). Final phage titres were confirmed using the standard plaque assay.

### 2.11. Storage Stability

To investigate the storage stability of encapsulated T3 in core-shell capsules, typically 1 g of capsules were placed in sealed 15 mL falcon tubes and stored in the fridge (4 °C) or at room temperature (~21 °C) for a period of 3 months. Three batches were produced and samples were taken after each month of storage, dissolved in Sorensen’s buffer as indicated above and the final phage titre confirmed using the standard plaque assay.

### 2.12. Statistical Analysis

IBM SPSS Statistics Version 25.0 was used for carrying out statistical analysis of the data collected. Sample averages were initially assessed for normal distribution of results using the Shapiro–Wilk test of normality. Data sets that were normally distributed were compared using 2-tailed *t-*tests (n = 5) with reporting of *p* < 0.05 as statistically significant.

## 3. Results

### 3.1. Characterisation of Core-Shell Capsules

**Figure 2:**
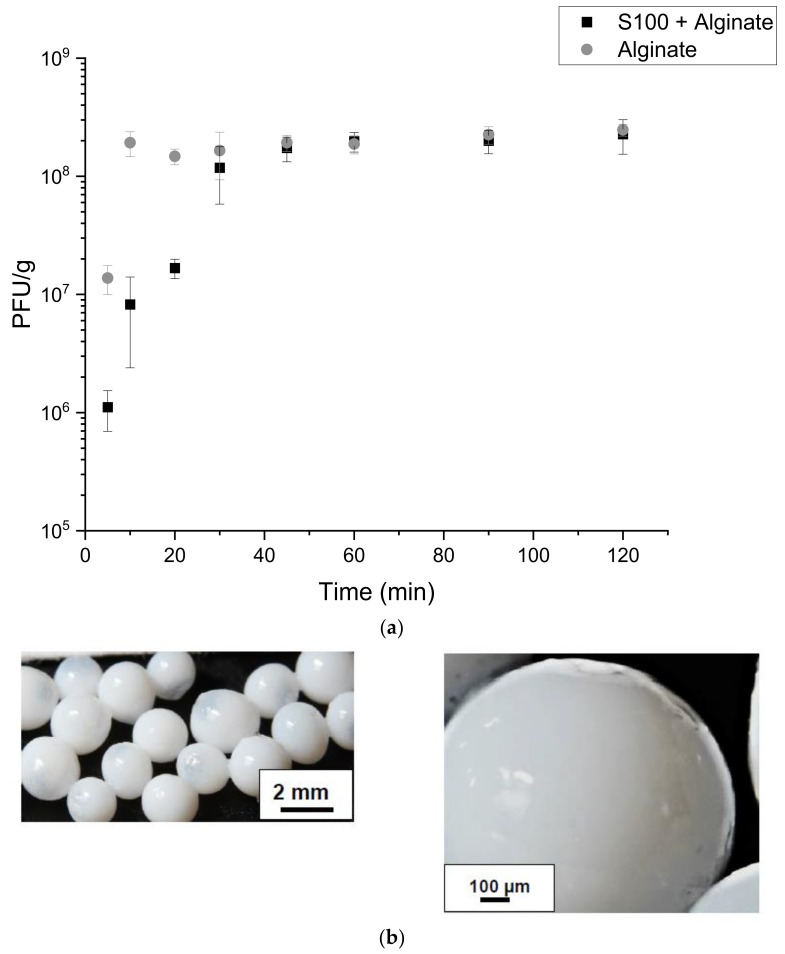

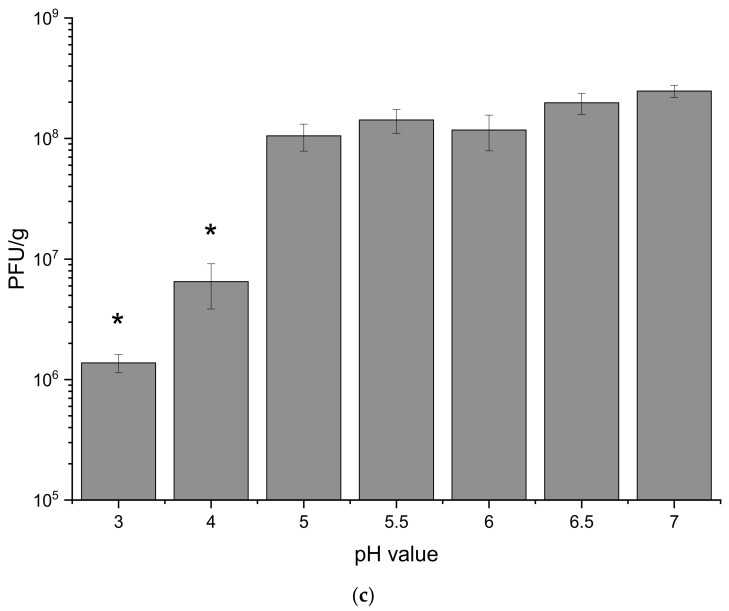
Release kinetics of phages from the core-shell capsules. Capsules were produced using a 200 µm core nozzle and 400 µm shell nozzle. The aqueous phase was loaded into a syringe pumping fluid to the shell nozzle and was composed of 10% (*w*/*v*) S100 and 1% (*w*/*v*) alginate or a solution of 1% (*w*/*v*) alginate only. The phage containing water-in-oil emulsion phase was loaded into a syringe pumping this phase to the core nozzle. The emulsion was produced using 10% (*v*/*v*) T3 phage stock in SM buffer, Miglyol with +5% (*v*/*v*) added PGPR. (**a**) Release kinetics of S100 + alginate and alginate only shelled capsules, 0.1 g of capsules were dissolved in 1 mL of Sorensen’s buffer (pH 7.5) and incubated for 2 h at 37 °C. At each time point, a sample was taken for phage enumeration using the plaque assay. Error bars represent one standard deviation. (**b**) Final core-shell particles after alginate crosslinking and polymer protonation, captured using a Nikon D7700 and a macro lens (Sigma 105 mm F2.8). (**c**) Phage release from alginate capsules in different pH buffers. 0.1 g of alginate only shelled capsules were incubated in 1ml of each buffer (Sorensen’s buffer) for 2 h, final phage titres were confirmed using the plaque assay.* Significant differences in phage titres (n = 4) using a paired *t*-test at each pH value compared to pH 7.5 (*p* > 0.05).

Core-shell capsules were produced using a concentric nozzle encapsulator system operated in a batch-production mode. Phage T3 suspended in an aqueous buffer solution was initially emulsified within an oil phase and then subsequently coated with a water phase containing S100 + alginate to provide the capsule outer shell. Final capsules appeared spherical and white in colour, due to the Eudragit S100 polymer, and were consistently 1–1.5 mm in diameter (Figure 2b). Optical images showed the capsules had a solid outer coating without any surface defects which otherwise could result in permeation of gastric acid or degradative gastric enzymes. A contiguous shell is important to prevent phage leakage outside of the core of the capsules unless dissolution of the shell occurred due to the pH change in the environment acting as a trigger for their release. A 200 µm inner nozzle equipped with a 400 µm outer nozzle allowed production of homogenous sized core-shell capsules. The flow rates were controlled at 12 mL/min for the ‘shell’ fluid and 4 mL/min and ‘core’ fluid respectively. The vibration frequency was set at 1000 Hz and an electrode voltage of 1000 V. Initial analysis confirmed these operating parameters provided a controlled droplet break-up, without capsule coalescence during manufacture. Initially, a concentration of 0.1 M CaCl_2_ was selected for alginate crosslinking immediately after production; however, slow crosslinking time caused phase separation of the beads and their aggregation. CaCl_2_ concentration was therefore increased 10-fold to 1 M CaCl_2,_ enabling rapid alginate hardening and gelation resulting in solid spherical capsules (Figure 2b). A volume of 100 mL of 1 M CaCl_2_ was selected to form the initial capsules as larger volumes required faster stirring rates to suspend the capsules, which often resulted in shear induced capsule breakage. The addition of 400 mL of 1 M CaCl_2_ after capsule formation ensured the alginate rich phase was saturated with Ca^2+^ ions to achieve complete crosslinking without capsule agglomeration.

Core-shell capsules were produced with either an outer shell composed of alginate only compared with a formulation that contained alginate and Eudragit to determine the advantages offered by using pH-responsive polymers (Eudragit) for targeted pH-triggered release. The impact of the formulations on capsule size, acid and heat stability and extrusion were investigated. Alginate-only capsules were smaller in size (~600 µm) (Table 1) and displayed elastic properties upon touch, compared to a firmer more rigid capsule upon addition of S100 to the formulation. These shell structural differences may indicate differences in the porosity of the shell responsible for conferring stability to the phages in the capsules when exposed to heat and shear stresses. The alginate capsules appeared to agglomerate slightly after the production process and gentle agitation was needed to separate the capsules. The slight agglomeration may influence the measured laser scattering size results and the CV value of 36%. However, visual observation confirmed that the alginate capsules were relatively uniform in size and shape.

Release kinetics of S100 + alginate capsules showed complete phage release was reached after 30–45 min of incubation (Figure 2a). 2 × 10^8^ PFU/g were encapsulated within the core-shell capsules with minimal phage losses through the production process. Alginate only capsules exhibited a more rapid release of phage cargo, with complete release occurring within 10 min of incubation at pH 7.5. Alginate capsules showed complete release of phage cargo at pH 5 (Figure 2c), with minimal (1%) release at pH 4. S100 + alginate capsules did not release encapsulated phages at pH 6.5 and below thereby allowing greater control of phage delivery in response to changes in environmental pH.

### 3.2. Stability of Free and Encapsulated Phage T3 Exposed to Simulated Gastric Conditions

**Figure 3:**
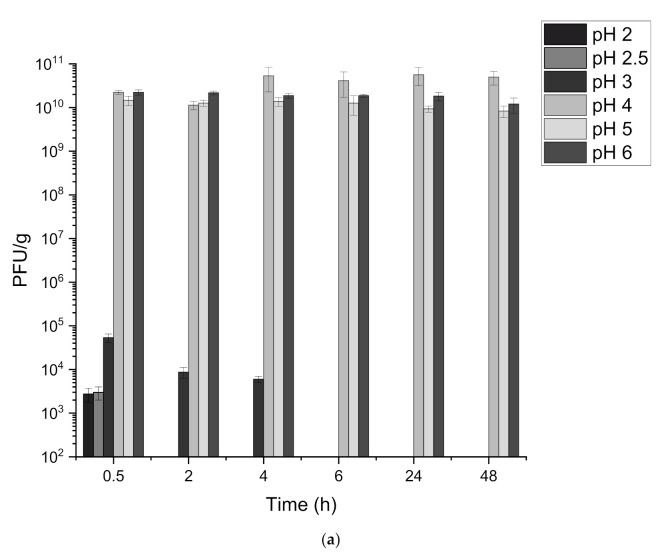

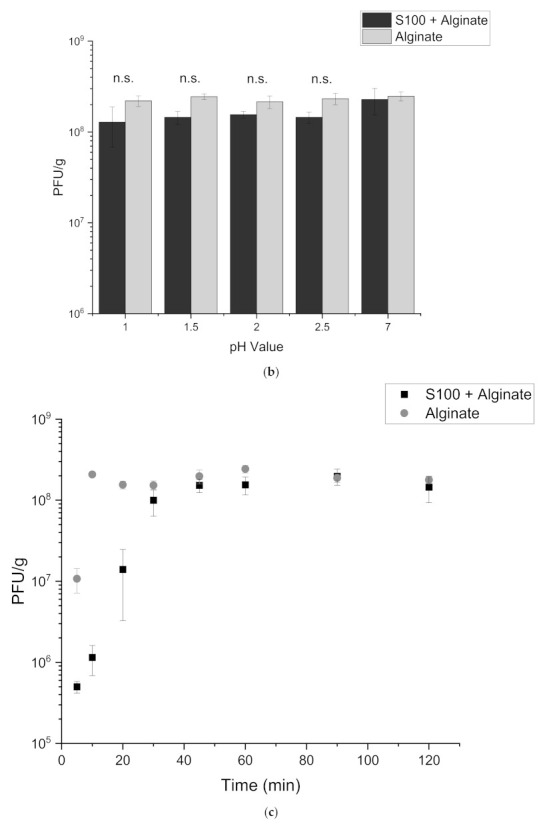
Acid stability of free phages and phages encapsulated in core-shell capsules. (**a**) Free phages were suspended in 0.2 M NaCl with pH adjusted to pH 2, 2.5, 3, 4, 5 and 6 (pH adjusted with HCl) at 37 °C for up to 48 h. Time points were taken throughout and the final phage titre enumerated using the plaque assay. (**b**) Core-shell capsules exposed to buffers at pH values 1, 1.5, 2 and 2.5. Capsules were suspended in 0.2 M NaCl buffer (adjusted with HCl) and incubated for 2 h at 37 °C. After this, the buffer was aspirated out and the capsules were dissolved in Sorensen’s buffer (pH 7.5). 3 batches of capsules were produced and average phage concentrations were calculated. (**c**) Release kinetics of core-shell capsules after exposure to pH 1, time points were taken for a 2 h period and phage titre enumerated using the plaque assay. Error bars represent one standard deviation. No significant difference (n.s.) in phage titres using a paired *t*-test at each pH value compared to no acid exposure (*p* > 0.05).

Initially, free T3 phages were assessed to confirm their resilience to acid exposure without encapsulation. T3 phages were exposed to a range of different pHs using buffered solutions from pH 1 to pH 6 for up to 48 h. No viable phages could be detected after 30 min at pH 1, 1.5, 2 or 2.5. Exposure to pH 3 did result in phage T3 survival for 4 h but significant losses ~7 log(10) were measured compared to the initial titre. T3 phages exposed to pH 4, 5 and 6 experienced minimal losses during the 48 h of testing (Figure 3a).

Core-shell capsules containing T3 were subsequently exposed to buffered acidic solutions to determine the level of acid protection offered to the phages by the S100/alginate matrix shell. No significant losses in phage concentration were recorded after 2 h of incubation in pH 1, 1.5, 2 and 2.5 (Figure 3b). The alginate-only capsules also provided excellent acid stability to the encapsulated phage, with no significant differences observed for any of the acidic buffers tested. To confirm the role of the oil in the water-in-oil emulsion providing phages acid stability, batches of capsules were produced using S100 + alginate as the outer shell and T3 suspended in SM buffer as the inner core. The S100 within the outer shell provided some acid stability, however significant phage losses were observed after acid exposure (Supplementary data, Figure S1). Phage inactivation occurred at pH 1 (2 log(10)) and pH 1.5 (2 log(10)). Acid exposure for 2 h at pH 2 and 2.5 did not show significant change in the final phage titre; this indicated that S100 provided some acid protection but phages in these capsules were less tolerant to acid exposure without the water-in-oil core.

The release kinetics of T3 after acid exposure was investigated to confirm whether further S100 protonation ensued which could affect the profile of phage release. Slightly slower release kinetics were observed for the 5 min and 10 min time points indicating some changes in the shell properties during acid exposure. However, complete release of T3 was still achieved within the 30–45 min of incubation in Sorensen’s buffer (Figure 3c). Alginate-only capsules were also tested for release kinetics after acid exposure, and no difference in the release profile was observed.

### 3.3. Thermal Tolerance of Free and Encapsulated Phage T3 at Extreme Temperatures

**Figure 4:**
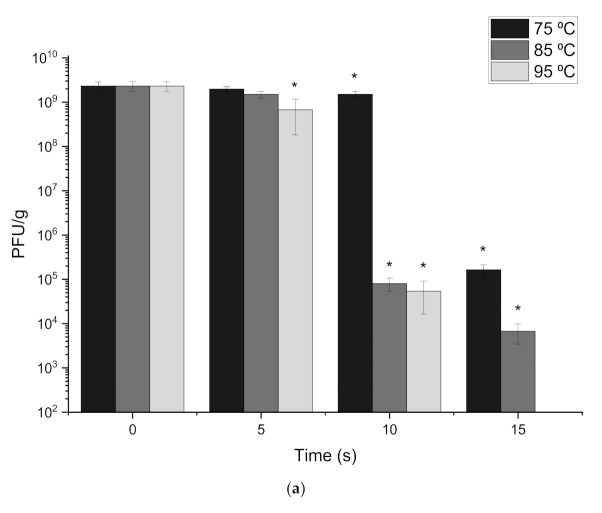

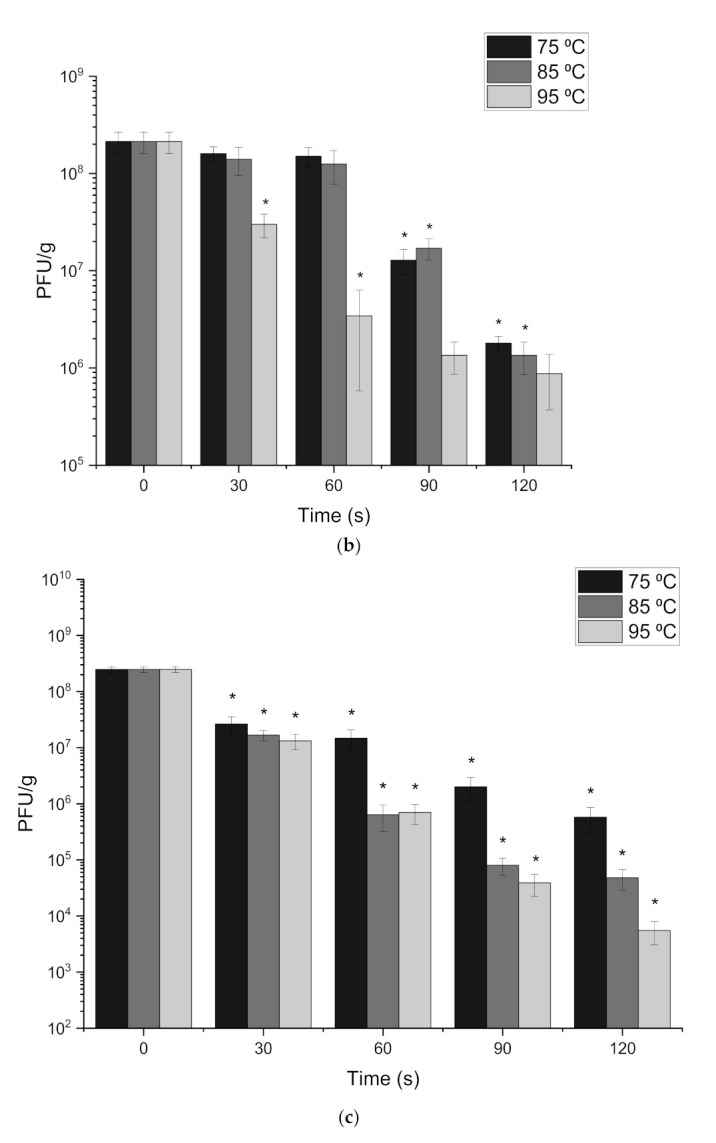

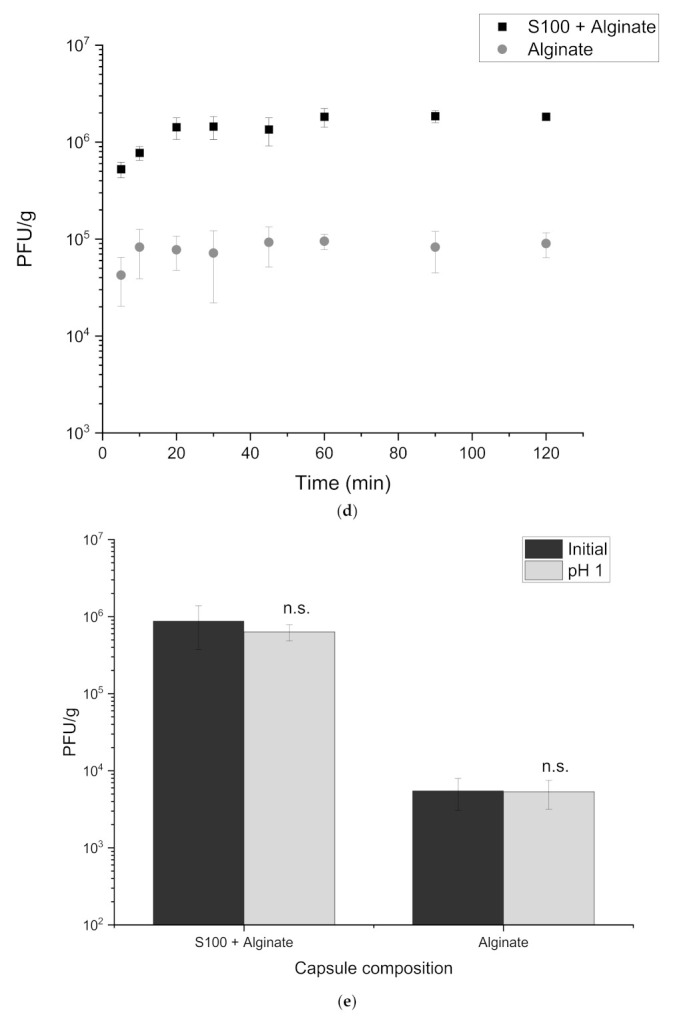
Heat stability of (**a**) free T3 phages, (**b**) S100 + alginate shelled capsules (**c**) alginate shelled capsules. Phages T3 in SM buffer were placed in a heating block at each temperature and samples were taken every 5 s until no viable phages could be detected. Typically, 0.1 g of capsules were weighed into metal crucibles and placed in a heating block set at 75 °C, 85 °C or 95 °C for 30 s, 60 s, 90 s and 120 s. After heat exposure, the capsules were dissolved in 1 mL of Sorensen’s buffer (pH 7.5) and phage titres enumerated using the plaque assay. 3 batches of capsules were produced, and 3 samples were taken from each batch to test heat stability. Averages of these results are displayed in the figure. (**d**) Release kinetics after exposure to 95 °C for 60 s, samples were taken at each time point and phage titres enumerated. (**e**) Acid exposure after 120 s at 95 °C. Samples were heated for 120 s then exposed to pH 1 for 2 h before release in Sorensen’s buffer (pH 7.5). Error bars represent one standard deviation. * Significantly different phage titres using a paired *t*-test at each time point compared to its previous time point (*p* < 0.05).

The effect of encapsulation on the thermal tolerance of phage T3 was investigated. Free phages were initially examined to determine how long phages can withstand heat stresses typically encountered during animal feed pelleting. Temperatures of 75 °C, 85 °C and 95 °C were tested, with results showing that no viable phages could be detected after 20 s of heating at each temperature. Exposure to 95 °C demonstrated significant phage loss after 5 s of thermal treatment and complete viable phage loss after 15 s. Significant phage losses were observed for 85 °C at 10 s and 15 s with a reduction from 2.3 × 10^9^ PFU/mL to 6.7 × 10^3^ PFU/mL. Similar results were obtained at 75 °C with significant phage loss observed at both 10 s and 15 s of thermal treatment, resulting in a final titre of 1.63 × 10^5^ PFU/mL. The rate of phage death was slowest at 75 °C and fastest at 95 °C (Figure 4a). Thus free T3 phages were not heat stable and could only withstand up to 15 s of thermal stress.

Encapsulated T3 phages were exposed to the same thermal treatment as free phages to determine the level of protection offered due to encapsulation. The rate of phage inactivation was highest when the core-shell capsules were heated to 95 °C. A significant difference in phage loss compared to the previous time point was observed after 30 s (*p* = 0.004) and 60s (*p* = 0.005) at 95 °C for S100 + alginate capsules, resulting in a 1 log(10) loss after 30 s. After 120 s of exposure to 95 °C, a final concentration of 8.8 × 10^5^ PFU/g was recovered. In comparison, at 75 °C and 85 °C phage titres were 1.8 × 10^6^ PFU/g and 1.3 × 10^6^ PFU/g respectively after 120 s (Figure 4b). To understand the role of the water-in-oil core against thermal inactivation of phage, capsules were produced with a S100 + alginate shell and T3 in SM buffer as the core. Upon heat exposure, the capsules without an oil-in-water emulsion core displayed complete T3 inactivation within 30 s at each temperature (75 °C, 85 °C and 95 °C) (data not shown).

Alginate only core-shell capsules provided less heat stability in comparison with the S100 + alginate capsules. Nevertheless, phages did survive exposure at each temperature over the 120 s exposure period. Significant differences were observed at each time point, compared to the previous time point. Thus, phage titres decreased significantly after each 30 s exposure interval. Final losses of 2 log(10), 3 log(10) and 4 log(10) were measured at 75 °C, 85 °C and 95 °C, respectively. Moreover, the final phage loss was over 2 log(10) more compared with the S100 + alginate capsules (Figure 4c). Nevertheless, T3 phages were able to withstand 120s of heat exposure when encapsulated in both formulations, compared to just 15 s of exposure before encapsulation.

Release kinetics of encapsulated T3 phages after thermal treatment were investigated to confirm the heating process did not affect the polymer release characteristics (Supplementary data, Figure S2). Capsules were exposed to 95 °C for 120 s and released using Sorensen’s buffer (pH 7.5). A burst release was still exhibited, as with the control sample, albeit at a lower titre due to the heat inactivation of the phage cargo in the capsules (1.8 × 10^5^ PFU/g). Complete release was achieved within 20 min of incubation for S100 + alginate capsules, a faster rate of release compared to control capsules. Alginate only capsules demonstrated no significant change in release kinetics after thermal treatment.

Acid exposure after heating confirmed the outer shell still conferred acid protection to the phages after thermal treatment. No significant differences were observed for either shell formulation after both heating and acid exposure (pH 1) in comparison to heating only. The capsule shell was, therefore, not adversely affected after 120 s exposure at 95 °C.

### 3.4. Simulated Extrusion Testing

**Figure 5:**
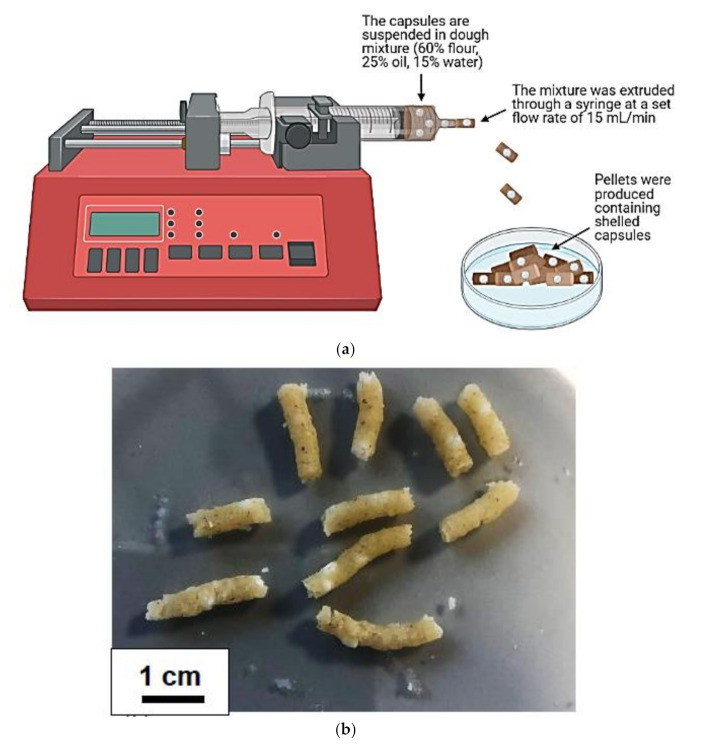

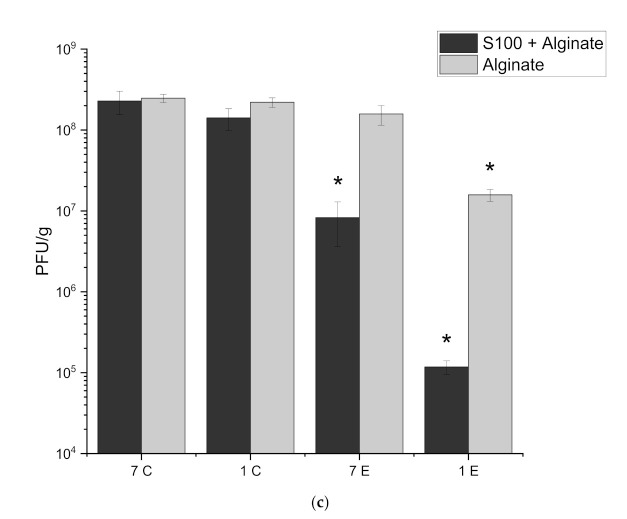
Extrusion of S100+ alginate and alginate only shelled capsules. (**a**) Core-shell capsules were suspended in an oil/flour mixture and extruded using a syringe at a set flow rate of 15 mL/min. (**b**) Pellets with a diameter of 3 mm and an approximate length of 1 cm were produced. (**c**) To determine capsule integrity after extrusion, capsules were harvested from the pellets and exposed to pH 1 buffer (0.2 M NaCl with pH adjusted using HCl) or dissolved using Sorensen’s buffer (pH 7.5). After acid exposure, the capsules were released in Sorensen’s buffer (pH 7.5). Phage concentration was measured using the plaque assay. ‘C’ and ‘E’ encode control and extruded capsules respectively. Error bars represent one standard deviation. * Significantly different phage titres using a paired *t-*test comparing extruded samples with the control samples.

Pellets were produced using a flour dough mixture and a syringe with a 3 mm outlet diameter (Figure 5a). Each pellet contained core-shell capsules; however, the capsules were not evenly distributed amongst each pellet (Figure 5b). After extrusion, the core-shell capsules were harvested from the pellets and their physical integrity was examined. Visually, the capsules appeared intact after extrusion as the size and shape had not changed. To measure if any capsules had burst during the extrusion process, capsules were exposed to pH 7.5 before and after extrusion. A significant reduction (*p* = 0.033) in phage titre was observed for S100 + alginate capsules, indicated by a 0.5 log(10) decrease, indicating some phage release from the capsules during pelleting. Alginate-only capsules showed no phage loss following extrusion when tested at pH 7.5.

To confirm whether the capsule shells were structurally damaged, they were exposed to pH 1 for 2 h before exposure to Sorensen’s buffer (pH 7.5). If the capsule shells were no longer contiguous, acid would enter and cause phage inactivation. A significant decrease (*p* = 0.0002) was confirmed for S100 + alginate capsules after this process with a 2 log(10) phage loss (Figure 5c). Thus, during extrusion the S100-alginate shell may have been damaged due to the shear stress experienced by the capsules adversely impacting acid protection afforded to the phage as a result. Extruded alginate-only capsules experienced a 1 log(10) reduction in phage titre after acid exposure which was considerably better compared with the S100 + alginate capsules. This suggested less damage to the alginate shell which was noted to be more pliable and, therefore, may be more resistant to shear induced damage during extrusion.

### 3.5. Storage Stability of Core-Shell Capsules

**Figure 6:**
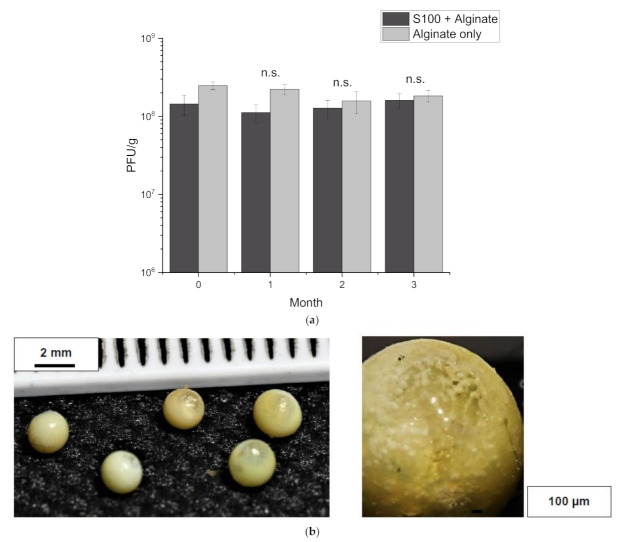
Storage stability of core-shell capsules. Capsules were stored dry in either refrigerated (4 °C) or room temperature (21 °C) conditions. (**a**) Monthly, samples were taken, and the phage titre was enumerated using the plaque assay. Error bars represent one standard deviation. * A paired *t*-test comparing each month to the initial phage titre) was completed, ‘n.s.’ refers to no significant difference (*p* > 0.05). (**b**) Discolouration of S100 + alginate shelled capsules stored at room temperature.

The storage stability of phages in the core-shell capsules was investigated to determine the shelf-life of the encapsulated phages. In refrigerated conditions (4 °C), no significant change was observed in phage titre over a 3-month storage period of testing for either of the two formulations (Figure 6a). Storage at room temperature conditions (21 °C), the phage titre in the S100 + alginate capsules decreased by 2 log(10) after 2 months. Furthermore, the appearance of the capsules changed to a yellow colouration, compared to the white colour of the shell initially (Figure 6b). This colour change did not occur in the refrigerated batch and consequently was attributed to occur due to the temperature of storage. Alginate-only capsules did not change in visual characteristics over the 3-month storage period stored at 21 °C. Although the capsules were stored dry, agglomeration did not occur, and capsules could easily be resuspended in buffer.

## 4. Discussion

Bacteriophage encapsulation for incorporation in animal feed pellets for animal biocontrol is an area that has received little attention to date in the literature. Animal food producers are facing challenges due to antibiotic resistance in bacteria and the need for antibiotic alternatives to enable effective control of infections in animals and associated potential economic losses e.g., due to *salmonella* carriage in chickens requiring culling of entire flocks of typically about 50,000 birds in the final trimester of a 42-day production cycle. Delivery of bacteriophages to animals through animal feed requires a feasible scalable method of encapsulation to protect the phages against different processing stresses including wet heat and extrusion as well as during transit through the animal digestive tract. The pH-responsive characteristics of the two formulations investigated in the present study could facilitate the targeted delivery of high phage doses to the site of infection in the lower GI tract in animals such as chickens and pigs. The utilisation of a commercially available concentric nozzle encapsulator enabled the rapid generation of uniform core-shell capsules containing phages suspended in a water-in-oil microemulsion. The core-shell particles fabricated here significantly improved phage protection against acid and thermal processing stresses. The production process offered flexibility in terms of using different formulations and the ability to produce capsules of varying sizes ranging between 100 µm->1 mm through the use of different sized nozzles. The capsule fabrication process operates at low shear rates, minimising damage to phages during capsule production, resulting in high phage yields in the final core-shell capsules.

Capsules produced using S100 + alginate were larger in size compared to alginate only capsules due to the increased viscosity of the S100 containing formulation. The higher viscosity of the formulation also resulted in a thicker shell around the oil droplet, thus producing larger capsules using the same operating parameters. The 200 µm inner nozzle and the 400 µm outer nozzle were selected to enable the continuous production of capsules. Use of smaller nozzles resulted in regular blockages and disruption of droplet formation. Using a combination of nozzles generated capsules approximately 1–1.5 mm in size with an average droplet size ~2× nozzle diameter [22]. Testing of nozzles with larger diameters (700 µm or 900 µm) produced larger capsules but these were fragile and the shell could easily break during handling and upon subjection to shear. The fabricated capsules made using each formulation appeared uniform in size and rapid crosslinking of the alginate shell using 1 M CaCl_2_ compared with 0.1 M CaCl_2_. Gentle agitation at 100 RPM during crosslinking and protonation of carboxylic groups prevented capsule damage during production. The white colouration of the S100 + alginate capsules provided visual confirmation of S100 polymer protonation upon exposure to pH 1.5 buffer for 2 h to form a rigid polymer shelled capsule. Alginate-only capsules appeared to release phages immediately upon exposure to pH 7.5, whereas S100 + alginate capsules required 30–45 min to achieve complete release. The smaller size of the alginate capsules may account for these results along with a thinner outer shell. The 1.5 mm S100 + alginate capsules had a significantly thicker shell requiring additional time for dissolution in the buffer. Previous literature reported phage-loaded alginate capsule beads 1.5 mm in size requiring up to 12 h incubation for complete release of the phage, but these capsules were solid capsules and not core-shell in structure [23]. The solid capsules were reported to show a sustained release of phages compared to the burst release observed for the core-shell capsules here (within 10 min). The formulations also differed with the concentration of alginate used (1% *w*/*v* as opposed to 2% *w*/*v*) which may affect the degree of crosslinking and the porosity of the alginate structure.

Eudragit methacrylate polymers are specifically manufactured for use in enteric coatings to protect encapsulated drugs and allow them to withstand gastric exposure in the human stomach. The dissolution of the S100-alginate capsules at pH 7 and above protected the phages from acid-induced damage. Unencapsulated phages were shown to be highly sensitive to acid, specifically at pH 2.5 and below when complete phage inactivation was noted which corresponds to pH values typically found in the gizzard and proventriculus of chickens. This observation highlighted the need for encapsulation to ensure high doses of phage T3 reach areas of the lower gastrointestinal regions infected with bacteria e.g., caeca of chickens. For the encapsulated phages, no significant losses in phage titre were observed after 2 h of acid exposure for the S100 + alginate beads, along with no change in the phage release kinetics. In comparison, a study conducted utilising membrane emulsification for the manufacture of S100 + alginate solid microcapsules reported no significant losses when exposed to pH 2 or 2.5, but a significantly longer time for complete release (5 h as opposed to 2 h) [24]. This could indicate that the solid S100 + alginate capsules prepared in that study were not fully protonated, thus the curing continued during incubation with the acidic buffers and formed a denser polymer matrix which resulted in increased dissolution times. Improving on previous published work, the significantly thicker S100 + alginate shell coating in the present study still exhibited a burst release mode of action after acid exposure with complete dissolution within a 30–45 min period. This suggested that protonation at 37 °C for 2 h was sufficient in terms of providing acid protection to the phages at values as low as pH 1 whilst still enabling a burst release mechanism to be maintained.

The use of an oil-in-water microemulsion in the core was shown to have a significant impact on the acid protection afforded to the phages in the core-shell capsules. This was confirmed by comparing phage titres recovered after acid exposure following production of S100 + alginate capsules without using the oil-in-water emulsion core. The acid tolerance of these capsules was significantly inferior compared with capsules formulated with the oil-in-water emulsion core. Significant losses in phage viability after simulated gastric acid exposure indicated the need for an oil-in-water emulsion core. The oil-in-water emulsion core may hinder H^+^ diffusion within the capsule core due to the hyrdophobicity of the oil phase thereby protecting T3 phages from acid induced inactivation. Eudragit S100 + alginate core-shell capsules did provide some acid protection to the T3 phages without the use of a water-in-oil emulsion core whereas the alginate only capsules produced without an oil-in-water emulsion core did not enable the T3 to withstand low pH acid exposure. The water-in-oil core formulation facilitated complete acid tolerance of the encapsulated T3 phages and presents a new, hitherto unexplored strategy for improving phage tolerance to acid exposure.

The use of alginate only for the shell composition similarly enabled protection of phages in the oil-in-water emulsion core. Insoluble alginate matrices are formed by ionotropic gelation using multivalent cations (Ca^2+^) [25]. The Ca^2+^ ions interact to form ionic bridges between polymer chains, facilitating acid stability of the final capsule. Alginate is commonly employed as a pharmaceutic delivery biopolymer due to its biocompatibility and safety for use in animals. Mammals cannot enzymatically degrade alginate as they do not possess alginase to cleave the polymer chains [25]. Specifically, alginate is employed for oral delivery of pharmaceuticals where shrinkage at low pH values protects the encapsulated material. At higher pHs above the dissociation pKa of the alginate carboxylate groups, diffusion of metal ions such as Na^+^ and K^+^ results in polymer dissolution and structural disintegration of the alginate shell releasing the encapsulated phage cargo e.g., in the upper compartments of the small intestine [25]. Several studies in the scientific literature have reported varying degrees of success in terms of stabilising phages and protecting them from acid damage in alginate microspheres. Encapsulation of *Salmonella enterica* subsp. *Typhimurium* phage Felix O1 in chitosan-alginate microcapsules resulted in increased survival of the phages compared with free phages, however increasing amounts of phage were inactivated as the pH was lowered (pH 2 and 2.5) and exposure times increased to 60 min [26]. Viable phages could not be detected after 60 min of incubation in pH 2.5 buffer, yet chitosan-alginate microcapsules provided complete acid protection with only a 0.5 log(10) loss. A different study where alginate microspheres were prepared encapsulating phage CA933P for lysis of enterohaemorrhagic *E. coli* concluded alginate offered no acid protection for the encapsulated phage after exposure to pH 1.6 [27]. A concentration of 3% (*w*/*w*) alginate was utilised and the beads were crosslinked in 500 mM CaCl_2_ for 12 h at 4 °C. Complete acid protection for T3 phages was shown in the present study upon exposure to a highly acidic solution (pH 1). The use of the oil-in-water emulsion in the core and the structure of the outer shell favourably impacted on the survival of the T3 phages in the core-shell capsules. A crosslinking time of 2 h at 37 °C in the present study provided greater acid protection compared with capsules produced using a crosslinking time of 12 h at 4 °C in literature which may be explained by slower rates of crosslinking at lower crosslinking temperatures. The thick shell layer surrounding the phage containing water-in-oil emulsion (Figure 1d) ensured that the encapsulated phages were protected from the acidic environment (~200 µm polymer shell layer).

The selection of the different pH-responsive shell polymers may allow delivery of phages to different compartments of the gastrointestinal tract. Complete dissolution of the alginate shell occurred at pH 5 and above. By comparison the S100 + alginate shell dissolved at pH 7 and above. Alginate shell dissolution tested at pH 4 showed minimal amounts of T3 phages were release from the core-shell capsules. With the S100 core-shell capsules, no release of T3 phages was detected even at pH values as high as pH 6.5. The pH responsive characteristics of S100 would enable phage delivery and release in the lower sections of GI tract in chickens and pigs which would not be achievable using alginate, as phage release would occur as soon as the intestinal pH reached pH 5 which is the case in the crop of chickens [28]. Different pH-responsive polymers within the Eudragit series (L100 and L100-55) could be utilised within this manufacturing process to deliver high phage doses to varying sections of the GI tract with alginate-based capsules providing a cost-effective commercially available alternative for lower-cost encapsulation [29].

The pelleting processes used in animal feed preparations may be a significant limiting factor in incorporating phages into animal feed products. Thermal stress protection for encaspulated enteric phages is an area that has not been investigated extensively. Results from the present study show that phages are easily inactivated at temperatures used in animal feed pelleting processes (75 °C–95 °C) [30]. Phage inactivation through thermal treatment has previously been tested on Lactococcal phages known to withstand pasteurisation temperatures. The phages were added to reconstituted skimmed milk (RSM) and exposed to 85 °C using a controlled heating block; the thermal stability of the 11 phages was found to vary considerably with some phages able to survive 30 min of heat exposure, whereas others were inactivated immediately [31]. Milk constituents are known to provide a protective medium for phages during thermal treatments. However, there is scant literature covering heat stability of enteric phages targeting pathogens such as *E. coli.* Thermal tolerance over a period of 120 s at 95 °C was afforded to T3 phages encapsulated in the core-shell capsules where an aqueous solution of T3 phages was emulsified in miglyol and PGPR in the core with an outer polymer shell. Both S100 + alginate and alginate only capsules provided heat stability at 95 °C for 120 s to phages which would otherwise be inactivated within 15 s without encapsulation. The shell was not damaged during the heat treatment confirmed by the acid exposure test (pH 1). The use of phase change agents with high thermal capacity may be a strategy to improve phage stability to thermal stresses. This would ensure that during a short period of exposure of the phages in the hot extrusion process, the phages are not inactivated. Core-shell beads with only an aqueous core resulted in phage inactivation and displayed poor heat tolerance. The S100 + alginate capsules appeared to provide more thermal tolerance in comparison to the alginate-only capsules. This may be due to the larger size of the S100 + alginate capsules resulting in a bigger thermal resistance which may have resulted in a lower temperature rise that the phages were exposed to inside the capsules. The heat stability afforded to phages in the core-shell particles with an oil-in-water core is a novel outcome of the study which requires further exploration and provides proof of principle for implementation of such a formulation in animal feed processing and capable of delivering phages to the GI tract in animals.

The shearing stress experienced by the capsules during the extrusion process resulted in capsule rupture. This could potentially release core contents from the core-shell capsules as well as making the phage vulnerable to acid exposure. Encapsulated phages themselves are known to survive compression stresses used in tablet manufacture with a recent paper on tableting of spray-dried Felix O1, a *S. Typhimurium* specific *Myoviridae*; powder was tableted by direct compression [32]. The direct compression process had no significant effect on phage survival. The spray dried particles were ~10 µm in diameter and therefore considerably smaller than the capsules produced in the present study. The smaller particle size of the spray-dried powders resulted in significant (1–2 log(10)) reductions upon exposure to SGF (pH 2). Capsule size is known to affect phage stability upon exposure to acidic pH [33]. Extrusion of capsules in the meal mixture appeared to cause more damage to the S100 + alginate capsules which were considerably larger in size and hence more vulnerable to shearing forces which may have resulted in breakage of the shell structure. The shell of the S100 + alginate beads was considerably more rigid and easier to fracture in comparison with the more elastic shell of the alginate capsules which may be better able to withstand the shear forces experienced during pelleting. Further work is needed to improve the resistance of the S100 + alginate core shell capsules to extrusion stresses including evaluation of smaller capsules incorporated in the animal feed meal.

The storage stability of phages in core-shell capsules produced with each formulation under refrigerated or room temperature conditions showed no significant phage losses for either formulation under refrigerated conditions over a 3-month storage period. However, loss in phage viability was observed for S100 + alginate capsules after 3 months stored at room temperature. Further work is needed to investigate the stability of the core-shell capsules incorporated in the animal feed after the pelleting process and stored under typical uncontrolled conditions encountered in the field.

## 5. Conclusions

Core-shell capsules encapsulating bacteriophage T3 in a water-in-oil emulsion in the core with either alginate only or S100 + alginate shell provided the phages with excellent protection upon acid exposure at pH 1 for 2 h. Furthermore, the encapsulated phages were significantly more stable in comparison with free phages upon exposure to thermal stresses up to 95 °C for 120 s typically encountered during pellet formation where extrusion of animal feed meal is carried out. The use of oil-in-water emulsified phages in the core of the core-shell particles could be a potential game changer in terms of using bacteriophages incorporated in animal feed pellets. Further research is needed to optimize such formulations including improving stability of the capsules to the pelleting process and the shelf life of the products. Furthermore, in vivo evaluation of the core-shell capsules incorporated in animal feed is needed with potential future applications including reduction of *salmonella* carriage in chickens and pigs.

## Figures and Tables

**Table 1:** Table outlining size characteristics of alginate only capsules. ImageJ was used for S100 + alginate capsules (n = 20). Laser diffraction was used for alginate only capsules. Error bars represent one standard deviation.

	S100 + Alginate	Alginate
Mean capsule diameter (µm)	1520	610
Standard deviation (µm)	110	236
Coefficient of variation (%)	7	38

## Data Availability

All relevant data are included in the manuscript. Raw data can be made available upon request from the corresponding author.

## Supplementary Materials

The following are available online at https://www.mdpi.com/article/10.3390/v13061131/s1, Figure S1: Acid stability of S100 + alginate capsules produced without an oil-in-water emulsion core. Capsules were exposed to acidic buffers (0.2M NaCl + HCl) for 2 h at 37 °C before release in Sorensen’s buffer (pH 7.5), Figure S2: Release kinetics of S100 + alginate core-shell capsules immediately after production (control), after pH 1 (acid exposure) for 2 h and after 120 s at 95 °C (heat exposure).
