# Supplementary material for: Bacteriophage Encapsulation in pH-Responsive Core-Shell Capsules as an Animal Feed Additive
Source: Viruses. 2021 Jun 11;13(6):1131. doi: 10.3390/v13061131 (PMC8231228; doi:10.3390/v13061131)
Supplement: Supplementary file 1 [file viruses-13-01131-s001.zip › viruses-1251468-supplementary.pdf]

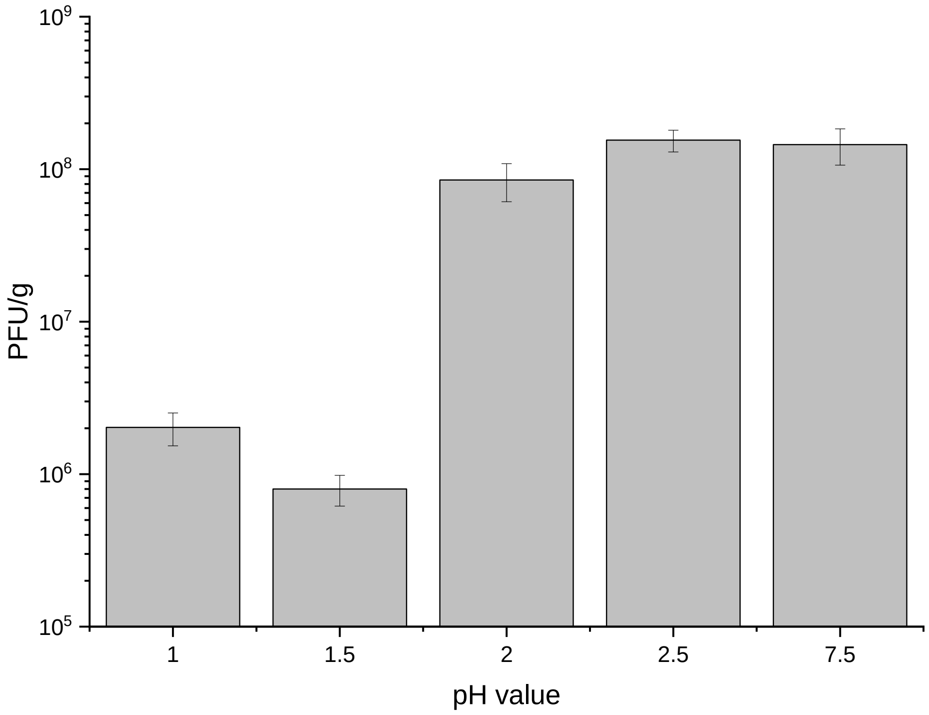

Figure S1. Acid stability of S100 + alginate capsules produced without an oil-in-water emulsion core. Capsules were exposed to acidic buffers (0.2M NaCl + HCl) for 2 h at 37 °C before release in Sorensen's buffer (pH 7.5).

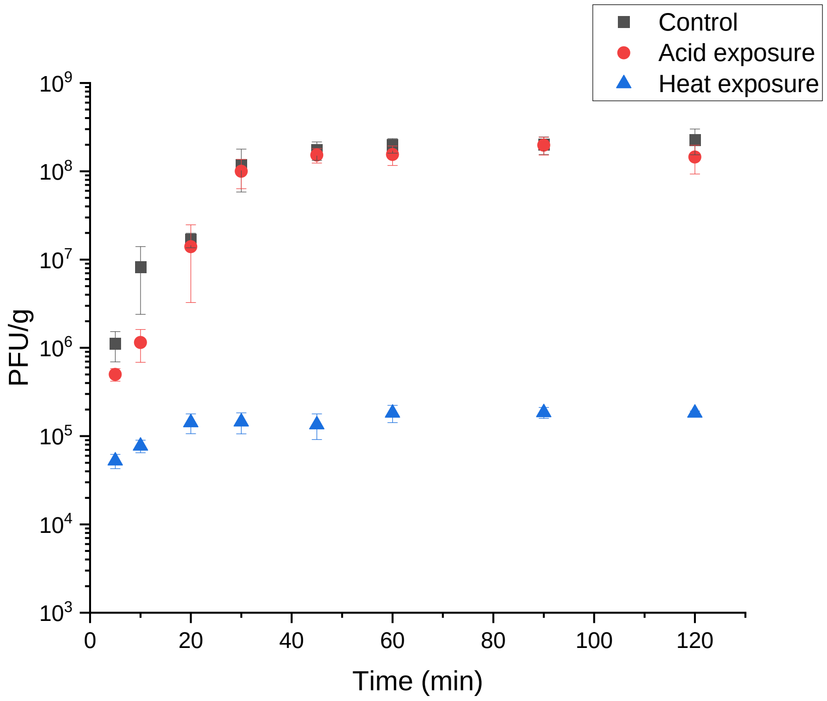

Figure S2. Release kinetics of S100 + alginate core-shell capsules immediately after production (control), after pH 1 (acid exposure) for 2 h and after 120s at 95 °C (heat exposure).
